# The role of amino acids on supramolecular co-assembly of naphthalenediimide–pyrene based hydrogelators†

**DOI:** 10.1039/c8ra00929e

**Published:** 2018-04-19

**Authors:** Srinivasa Rao Nelli, Rajan Deepan Chakravarthy, Mohammed Mohiuddin, Hsin-Chieh Lin

**Affiliations:** Department of Materials Science and Engineering, National Chiao Tung University Hsinchu 300 Taiwan Republic of China hclin45@nctu.edu.tw

## Abstract

This report describes the two component self-assembly of π-capped amino acid hydrogelators (serine (S), aspartic acid (D), glutamic acid (E) or lysine (K)) prepared from pyrene (Py) based donor and naphthalenediimide (NDI) based acceptor molecules. The co-assembly can be triggered to form hydrogels by varying the pH conditions and the major driving forces behind the hydrogelation were found to be the formation of a strong charge-transfer (CT) complex and hydrogen bonding interactions at suitable pH conditions. The NDI–Py blends with matched donor/acceptor amino acid pairs undergo self-assembly under acidic pH conditions, whereas the blend (NDI–S + Py–K) with a mismatched amino acid pair forms a stable hydrogel under physiological pH conditions. UV-Vis, FTIR and rheological studies clearly indicate the formation and the stability of these CT-induced hydrogels. These hydrogels are of nanofibrous morphology with an average diameter of about 6–9 nm as evidenced by TEM analysis. In addition, this novel NDI–Py mixed component system exhibited good biocompatibility towards PC3 cells. Overall, since hydrogels based on CT-mediated two-component assemblies are very rare, our newly discovered NDI–Py hydrogels provide chemical insights into the design of a CT-induced hydrogelator and might facilitate various applications in biomedical engineering.

## Abstract 2

The importance of side chains of amino acids for NDI–Py co-assembled hydrogelation is described.

## Graphical abstract

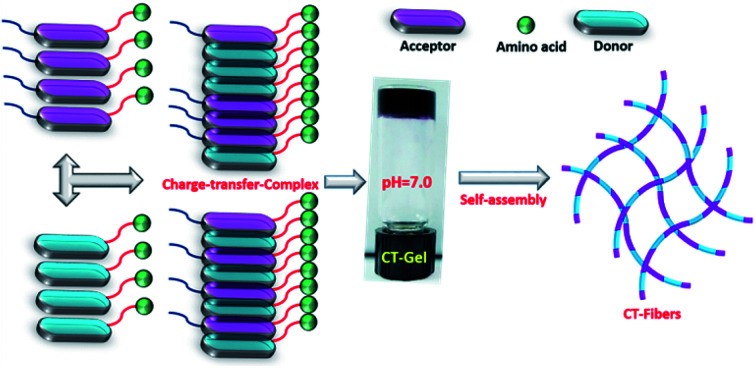

## 1. Introduction

Supramolecular assemblies, prepared from π-conjugated building blocks have attracted great attention owing to their diverse range of material applications.^1–4^ Among them, aromatic peptide/amino acid based supramolecular nanomaterials have received significant interest in recent years due to their potential applications in tissue engineering, drug delivery, enzyme assay and protein separations.^5–9^ Particularly, hydrogels prepared from such organic materials possess significant bio-functionality and biodegradability. Such molecules aggregate in aqueous conditions through various weak non-covalent interactions through hydrogen bonding, hydrophobic interactions and π–π interactions to generate one-dimensional (1D) nanofibers. These 1D fibers, elongated into the three-dimensional (3D) space yield 3D networks that are capable of entrapping a large amount of water molecules. Similarly, co-assembly of binary organic scaffolds with donor and acceptor π-electron moieties are known to undergo facile ground state charge transfer (CT) complex formation, which result in alternatively/cofacially organized aromatic molecules.^10^ Such two component CT based materials have been studied in several system including supramolecular gels, rotaxanes, catenanes and synthetic ion channels.^11–15^ Hydrogels based on CT-mediated two-component assemblies are rare in the literature. The physical and chemical properties of two-component gels are incredibly different from single-component ones. Although few supramolecular co-assembled systems are known in literature, there is still limited understanding about the structural and functional properties of such gels.^16–22^ This necessitates the development of new CT based multi-component supramolecular hydrogels.

**Scheme 1:**
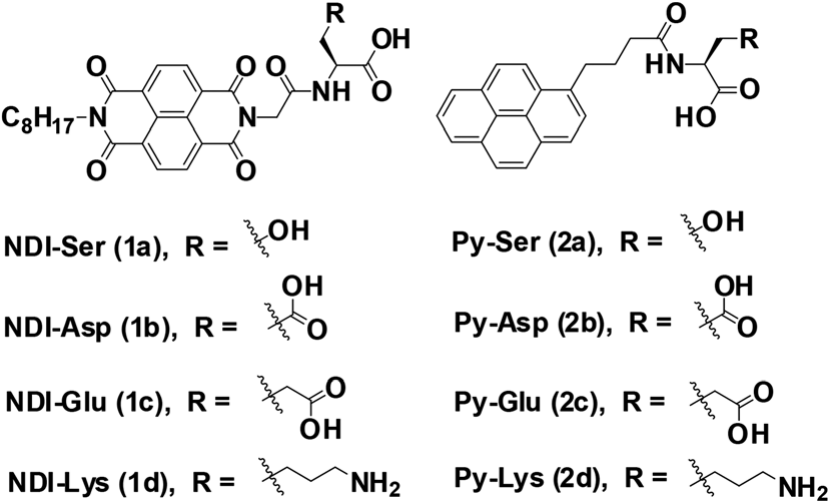
Molecular structures of NDI/single amino acids (1a–1d) and Py/single amino acids (2a–2d).

Herein, we explore the co-assembly properties of a series of pyrene (Py) and naphthalenediimide (NDI) based donor–acceptor systems. NDI and Py derivatives are the promising organic π-conjugated building blocks, which can be used as acceptors and donor scaffolds, respectively, to create such CT-mediated assemblies. Few reports have shown that the formation of supramolecular hydrogels is from self-assembly of CT-complex.^23–28^ For example, Ulijn *et al.* developed a fully reversible thermolysin-catalyzed peptide based functional dynamic combinatorial libraries in the hydrogel phase based on the self-assembly of NDI–amino acid/dipeptide amide bio-conjugates.^23,24^ Bhattacharya *et al.* have demonstrated the formation of CT-induced hydrogels with NDI and Py systems.^25,26^ In another interesting study demonstrated by Hu *et al.* described CT-induced supramolecular hydrogels by Py and 2,4,7-trinitrofluorenone systems.^27^ In our recent report, we demonstrated the co-assembly of a series of NDI/amino acids with Py butyric acid at acidic pH condition.^29^ As a next step, we continue our efforts towards the development of CT-based hydrogels formed under neutral pH. In this study, we prepared a series of asymmetric NDI–capped amino acids (NDI–serine (1a), NDI–aspartic acid (1b), NDI–glutamic acid (1c), NDI–lysine (1d)) and Py conjugates (Py–serine (2a), Py–aspartic acid (2b), Py–glutamic acid (2c), Py–lysine (2d)) as shown in Scheme 1. We investigate whether NDI–Py structures can form CT-based supramolecular hydrogels at neutral pH condition. Detailed investigation suggested that NDI–Py amino acids systems self-assembled in aqueous medium through strong CT-complex formations along with additional hydrogen bonding interactions. Furthermore, the NDI–Py system showed low cytotoxicity in human prostate cancer cells (PC3), thus making it a suitable carrier for drug delivery applications.

## 2. Experimental

### 2.1 Synthesis

All starting materials and solvents were obtained from commercial sources and used as received. The synthesis of NDI and Py capped amino acids were performed by solid phase peptide synthesis (SPPS) using classical Fmoc protocol. The purity of the final compounds was confirmed using ^1^H-NMR, ^13^C-NMR, and MS techniques. NMR spectra of the final peptide compounds were recorded at 300 MHz using Varian Unity Inova NMR instrument in deuterated solvents. High resolution mass spectra were recorded on Micromass Q-TOF MS spectrometer.

### 2.2 Gelation studies

Hydrogels were prepared by weighing suitable amount of the compounds in a screw-capped 2 mL vial (diameter: 10 mm). Aqueous NaOH solution was added and the resulting solution was vortexed for 5 s and sonicated for few minutes to get a clear solution. Then pH and concentration of the solution were carefully adjusted to trigger the hydrogelation. After that, the sample was allowed to stand at room temperature for few hours to complete the self-assembly process. Gelation was considered to have occurred when a solid-like material obtained did not exhibit any kind of gravitational flow (vial inversion test) during a period of 2–5 min in each case. Temperature *T*_gel–sol_ was measured using circulating water bath (digital temperature control) by increasing the temperature of the hydrogel at the rate of 1 °C min^−1^.

### 2.3 Transmission electron microscopy

TEM images were captured using a Hitachi HT7700 transmission electron microscope operating at an accelerating voltage of 100 kV. The sample was prepared by dropping a small amount of hydrogels (10 μL) directly onto 200 mesh carbon-coated copper grids. Excess amount of the hydrogel was blotted by capillary action using filter paper. Negative staining was applied using uranyl acetate (2% w/v) and the mixture was again blotted to remove excess stain. The sample was then allowed to air-dry for 2 days before the images were collected.

### 2.4 Rheological tests

Dynamic rheological measurements were carried out using TA Discovery rheometer (DHR-1) using 20 mm parallel plate geometry. The hydrogel sample (200 μL, 3 wt%) was sandwiched between stainless parallel plate geometry and a stationary bottom plate and the sample was allowed to stand for 2 hours before taking rheological measurements. The storage modulus (G′) and loss modulus (G′′) were measured as a function of the frequency between the range of 0.1 rad s^−1^ and 100 rad s^−1^ at strain of about 0.8%.

### 2.5 Spectroscopic studies

UV-Vis spectra were recorded on a HP8453 UV-Visible spectrophotometer. Circular Dichroism (CD) experiments were carried out on a JASCO J-810 spectrometer. Fourier transform infrared spectroscopy (FTIR) measurements were performed using a Perkin-Elmer spectrophotometer.

### 2.6 Cell viability tests

Biocompatibility assay of NDI–Py amino acid conjugates was measured through MTT cell viability test. PC3 cells (CRL-1435, ATCC) were chosen as cell model for this study. The cells were pre-incubated into 24-well plates at a density of 50 000 cells per well with 0.5 mL medium (DMEM) containing 10% FBS and 1% Penicillin–streptomycin solution and incubated for 24 h. Then the culture medium was replaced with fresh medium. Samples prepared at different concentrations (10, 50 and 100 μM) were added then cells were placed into solution and incubated for 24 and 48 h respectively. After that, the fresh medium supplemented with 0.5 mL of MTT reagent (4 mg mL^−1^) was added per well and the cells were incubated for another 4 h. Then the medium containing MTT was removed and DMSO (0.5 mL per well) was added to dissolve the formazan crystals. Each 24-well was transferred to 96 well plate. The optical density of the resulting solution was measured at 595 nm, using an absorbance micro plate reader (Infinite F50, TECAN). Cells without the treatment of the compounds were used as the control. The cell viability percentage was calculated by the following formula: the cell viability percentage (%) = OD (sample)/OD (control).

## 3. Results and discussion

### 3.1 Design and synthesis of NDI and Py derivatives

**Scheme 2:**
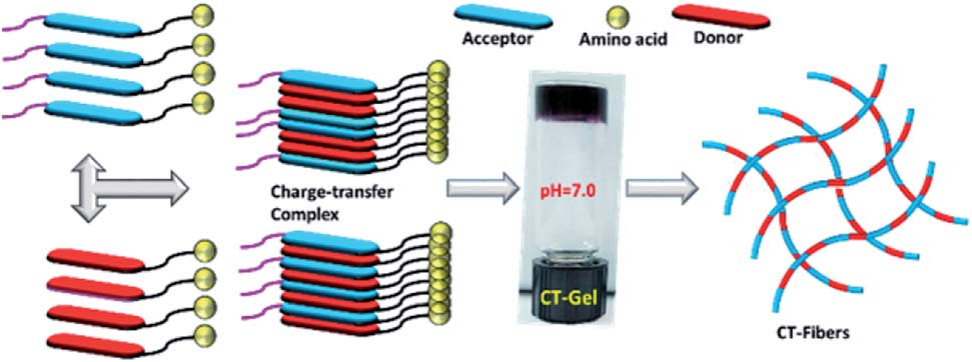
Plausible mechanism for the formation of CT-based hydrogel for NDI–S and Py–K.

To develop CT-based supramolecular hydrogels, we have synthesized a small library of NDI (1a, 1b, 1c, and 1d) and Py (2a, 2b, 2c and 2d) capped amino acid conjugates (Scheme 1) based on the method described by our previous report.^30^ Amino acids with different chemical functionalities have been used for this study to generate various non-covalent interactions during self-assembly. For example, π-conjugated compounds with serine residue facilitate hydrogen bonding and compounds with aspartic acid, glutamic acid or lysine residue generate additional electrostatic interactions during hydrogelation process.^31^ Long chain alkyl group is generally attached at the other end of NDI group to facilitate the aggregation of NDI conjugates in aqueous medium as described in our previous publication.^32,33^ The NDI–Py blends with matched amino acid pairs self-assembled at acidic pH conditions, whereas the blend (NDI–S + Py–K) with mismatched amino acid pair formed stable hydrogel at physiological pH condition (Table 1). The schematic mechanism of CT induced hydrogelators is shown in Scheme 2. The CT interactions between donor (2a–2d) and acceptor (1a–1d) molecules were studied by various spectroscopic techniques.

**Table 1:** Correlation matrix with different blend conditions tested in the study. Main diagonal entries are blends with matched amino acid pairs and off-diagonal entries are those with mismatched amino acid pairs

NDI or Py^a^^,^^b^	Py–S (2a)	Py–D (2b)	Py–E (2c)	Py–K (2d)
NDI–S (1a)	**OS**	OS	OS	OG pH = 7.0
NDI–D (1b)	OS	**OS**	OS	OS
NDI–E (1c)	OS	OS	**OS**	OS
NDI–K (1d)	OS	OS	OS	**OVS**

a OG: opaque gel, OS: opaque solution, OVS: opaque viscous solution.

b All pairs studies were carried out at neutral pH condition.

### 3.2 Gelation properties and nanofibrous morphology

We begin our experiment by mixing structurally related NDI and Py capped amino acids in aqueous condition (1 : 1 ratio) at different concentrations and the self-assembly is initiated by varying the pH of aqueous solution. The formation of hydrogels was confirmed by using inverted tube method (see ESI†). The mixtures 1a + 2a, 1b + 2b and 1c + 2c resulted in hydrogel formation at pH 3.5, 3.3 and 3.6, respectively (Table 2). In contrast, the compound of 1d + 2d formed a stable hydrogel at pH 6.3. All the four blends exhibit deep purple colour due to the formation of characteristic CT complex (Fig. S1, ESI†).^34,35^ However, we did not observed any hydrogelation at neutral pH condition. We then evaluate self-assembly behavior of all structurally mismatched NDI–Py amino acid systems at neutral condition. As shown in Table 2 the mixture 1a + 2d forms a stable hydrogel at 3 wt%. The 1 : 1 mixture 1d + 2a, on the other hand, afforded viscous solution under similar condition. There are three possible reasons for this pH dependent hydrogelation behavior. One is that electrostatic interactions between NH_3_^+^ and COO^−^ groups of lysine residues as in the case of 1d + 2d. Such interactions are highly facile at neutral or mild acidic condition which may lead to interlocked molecular arrangement during self-assembly. The ionic interactions in combination with various other non-covalent interactions could result in supramolecular hydrogelation. The second is that negatively charged COO^−^ group of aspartic or glutamic acids moieties, resulted in repulsive electrostatic interactions between the molecules at neutral pH condition. When the solution has lower pH value, such amino acid residues (Asp, Glu) may have neutral charge, leading to supramolecular self-assembly through hydrogen bonding network. Finally, the hydrogen bonding ability of serine residue is in the case of 1a + 2a. Serine is a polar hydrophilic neutral amino acid, can form hydrogen bonding at neutral pH. However the mixture 1a + 2a did not lead to any hydrogelation at a pH of 7.0. This may be due to the formation of lower order aggregates. Lowering the pH to 3.5 resulted fibrillar network formation which could entrap large solvent molecules leading to supramolecular hydrogels. We compared the hydrogelation condition of individual NDI and Py components to understand the self-assembly of mismatch pair (Table S1†). In the case of mixture 1a + 2d, both independent components could form stable hydrogels at suitable pH conditions *i.e.*, NDI–S (1a) forms stable gels at pH 4.5, whereas Py–K (2d) forms opaque gels at basic pH 9.0. So, the dual component hydrogels could be resulted upon mixing of 1a and 2d. However, in the case of 1d + 2a binary system, the donor component Py–S (2a) could not generate any hydrogel independently over the entire pH range (2–12). Such behaviour may also play a role co-assembly of NDI–Py systems. Similarly, the hydrogelation was not noticed for other mismatched combinations. All these blends, including 1d + 2a, may form hydrogels at acidic pH conditions, however, that is not the primary focus of this study.

**Table 2:** Physical properties of 1 : 1 blend of NDI–Py mixed compounds at 3 wt%

Entry	pH	Appr.^a^	*T*_gel–sol_ (°C)	Fiber width (nm)
1a + 2a	3.5	OG	35	6.1 ± 0.5
1b + 2b	3.3	OG	40	6.6 ± 0.5
1c + 2c	3.6	OG	40	8.6 ± 0.8
1d + 2d	6.3	OG	45	7.6 ± 0.4
1a + 2d	7.0	OG	40	6.1 ± 0.7
1d + 2a	7.0	OS	n.d.	n.d.

a OG: opaque gel, OS: opaque solution, n.d.: not determined.

**Fig. 1:**
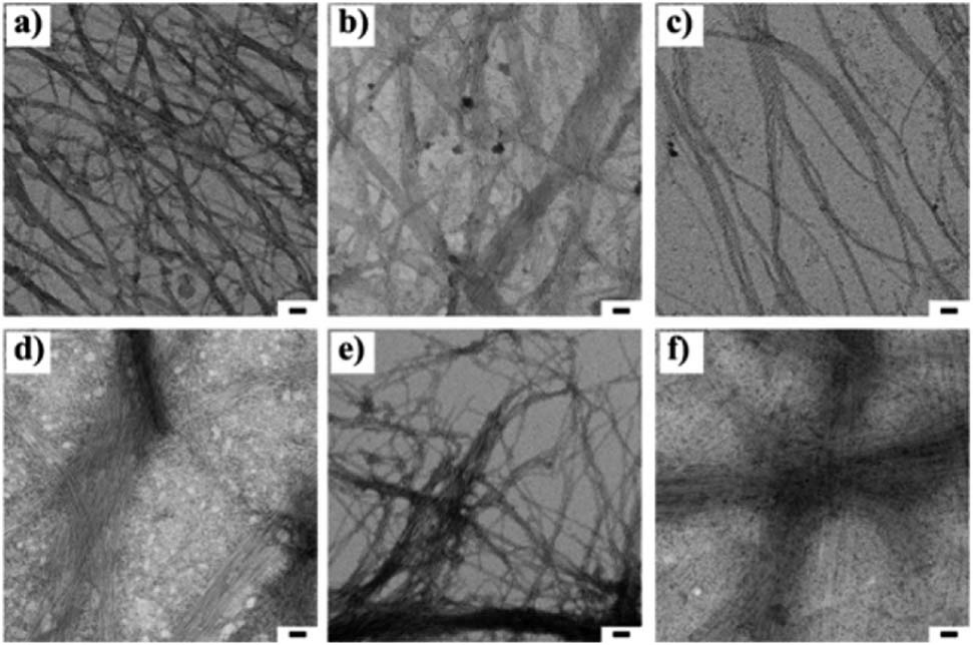
Transmission electron microscopic images of 1 : 1 blend of (a) 1a + 2a, (b) 1b + 2b, (c), 1c + 2c, (d) 1d + 2d (e) 1a + 2d and (f) 1d + 2a at 3 wt% respectively. The scale bar indicates 50 nm.

The nano-sized morphology of the hydrogels was examined by using transmission electron microscopy (TEM). The corresponding TEM images of 1a + 2a, 1b + 2b, 1c + 2c and 1d + 2d show the morphology of well-ordered self-assembled nanofibers at 3 wt% (Fig. 1a–d) in aqueous media. From the TEM analysis, we observed compounds 1a + 2a, 1b + 2b, 1c + 2c and 1d + 2d self-assemble into nanofibrous network with the average diameter of 6.14 ± 0.5 nm, 6.56 ± 0.53 nm, 8.58 ± 0.82 nm and 7.55 ± 0.4 nm, respectively, at 3 wt% whereas the diameter of mismatched system (1a + 2d) is 6.08 ± 0.73 nm (Fig. 1e). CT interactions may play a significant role in the formation of different nanofibrous morphology and TEM data suggested that our compounds were capable of forming nanofibrous networks.^35^ No clear morphological features were observed for 1d + 2a (Fig. 1f). To test the viscoelastic property of CT-based hydrogels, we performed the rheological measurements with the mode of dynamic oscillation frequency (0.1–100 rad s^−1^) at room temperature. All the co-assembled hydrogels are elastic materials since the observed storage moduli (G′) were higher than their loss moduli (G′′). In Fig. S2,† it is clear that blend 1d + 2d had the largest G′ of about value 100 Pa and the blend 1a + 2a exhibited the lowest G′ value. We further investigated the stability of hydrogels by measuring gel-to-solution transition temperature (*T*_gel–sol_) of each gel materials. The *T*_gel–sol_ at 3 wt% for 1a + 2a, 1b + 2b, 1c + 2c, 1d + 2d and 1a + 2d were found to be all above 35 °C (Table 2). Therefore, all these blends exhibited similar stability.

### 3.3 Spectroscopic analysis

**Fig. 2:**
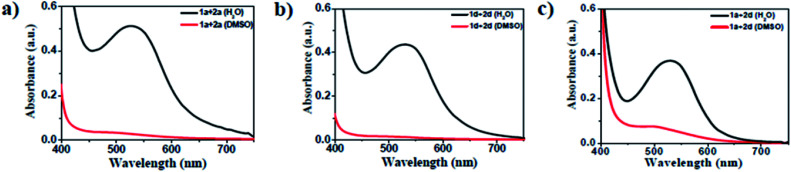
UV-Vis absorption spectra of the 1 : 1 blend of (a) 1a + 2a, (b) 1d + 2d and (c) 1a + 2d (black in H_2_O and red in DMSO) at 20 000 μM.

**Fig. 3:**
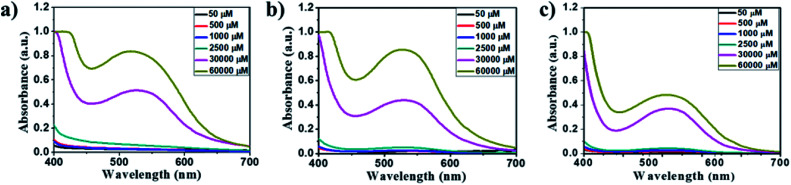
Concentration-dependent UV-Vis absorption spectra of the 1 : 1 blend of (a) 1a + 2a, (b) 1d + 2d and (c) 1a + 2d at 50–60 000 μM in aqueous media.

**Fig. 4:**
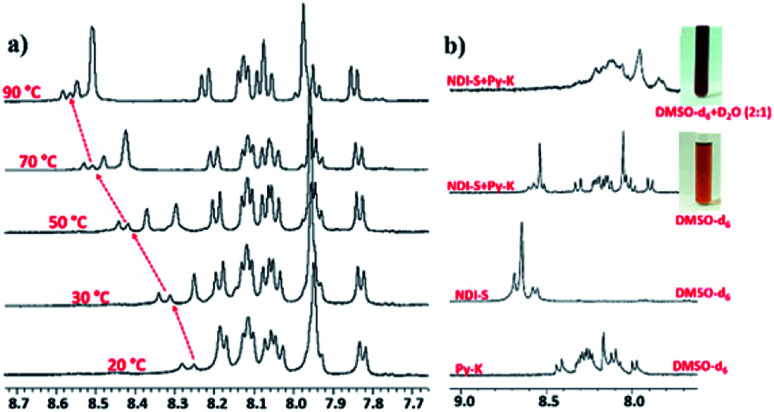
(a) Temperature dependent ^1^HNMR spectra 1a + 2d in DMSO-d_6_ : D_2_O (2 : 1); (b) solvent dependent ^1^HNMR spectra of 1a, 2d and 1a + 2d. Inset images showing the colour of 1a + 2d mixture in different solvents.

The amino acid group of hydrogelators greatly influences the structural morphology and stability of the hydrogels. By using various spectroscopic techniques, we examined the non-covalent interactions of two-component self-assembled NDI–Py compounds. Fig. 2 and S3,† show solvent-dependent UV-Vis absorption spectra of the blends in water and dimethyl sulfoxide (DMSO) conditions measured in the range of 400–800 nm. In the case of water, a strong CT signals with the absorption maximum at 540 nm appeared and the intensity further increased with increase in the concentration clearly indicating the formation of self-assembled system through strong π–π stacking and hydrogen bonding (Fig. 3 and S4†). The thermal stability of the CT-complex was determined using temperature-dependent UV-Vis absorption and all the co-assembled hydrogels were found to be stable in the range 20–90 °C. The intensity of CT band gradually decreased with increasing temperature suggesting that CT interactions of molecules were mitigated at high temperature (Fig. S5a–e†). A very weak CT signals was observed for the complex in DMSO condition, which may be due to the poor aggregation state of the system. CD spectroscopy is a powerful method for investigating the relative intermolecular orientation within the assemblies.^36,37^ The CD spectra of all NDI–Py (Fig. S6a–e†) blends showed a negative excitonic cotton effects at around 220 nm which is typical for n → π* transition of hydrogen bonded carbonyl groups.^36^ In addition, we observed peaks at around 240, 280, 360 and 380 nm could be attributed to absorption of NDI and Py groups.^37^ The CD spectrum of blend 1a + 2a displayed a bisignated cotton effect at 390 nm (Fig. S6a†). Mixtures of 1b + 2b and 1c + 2c shown positive Cotton effect at around 350 nm, whereas the mixtures 1d + 2d and 1a + 2d exhibited negative Cotton effects at the same spectral region which may be due to different chiral orientation of self-assembled aggregates. Thus, UV-Vis absorption and CD spectra clearly demonstrate the intermolecular interactions and orientation of the π-conjugated chromophoric units in aqueous conditions. To gain further insight into the NDI and Py interactions, temperature and solvent-dependent ^1^H-NMR studies were performed on the mixture of 1a + 2d. The aromatic proton signals of 1a + 2d system in DMSO-d_6_ : D_2_O (2 : 1) showed moderate downfield shift when the temperature increased from 20 to 90 °C. These spectral changes are possibly due to the disassembly of CT complex at high temperature (Fig. 4a–b). Furthermore, the aromatic signals of 1a + 2d in DMSO-d_6_ : D_2_O at 90 °C coincided with the sample in pure DMSO-d_6_, suggesting the monomeric state.

**Fig. 5:**
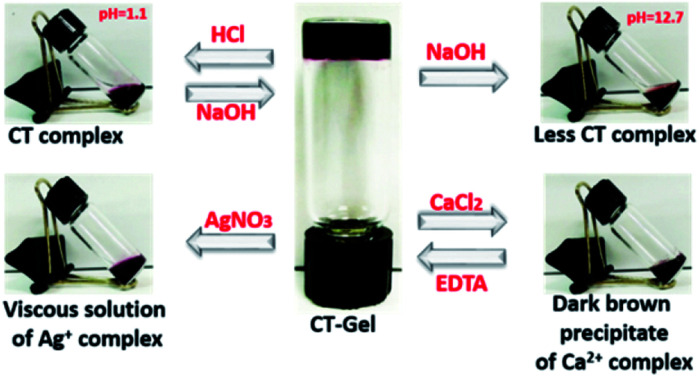
Responsive behaviour of 1a + 2d hydrogel under various condition.

Solvent-dependent FTIR spectra of blending compounds can be used to identify the secondary structures of the self-assembly nanostructures in hydrogels (Fig. S7 and S8†).^38–41^ The FTIR spectrum of NDI–Py blend in aqueous medium showed a strong absorption band below 1620 cm^−1^ often correlated to the formation of fibrillar aggregates.^41^ On the other hand, DMSO samples showed very weak signals at this region. In addition, the two amide carbonyl peak appeared at 1660 cm^−1^ and 1700 cm^−1^, which correspond to their symmetric and asymmetric carbonyl stretching frequencies, respectively. X-ray diffraction (XRD) were performed to understand the molecular packing of the materials. The XRD spectra of lyophilized NDI–Py samples showed a strong signal at around 2*θ* = 26° (*d* = 3.44 Å) which is the characteristic peak for the π–π stacking of aromatic moieties and a signal at 2*θ* = 11.21° (*d* = 7.71 Å) indicating the lamellar type arrangement of NDI–Py conjugates (Fig. S9†).^42–44^ Next, we analysed the morphological transition of the hydrogels with various chemical stimuli. The hydrogel 1a + 2d show intense purple colour in aqueous solution due to the formation of CT-complex at neutral pH. Addition of few drops of 6 N HCl, resulted in complete disruption of hydrogel matrix. However, no visible colour change was noticed. The hydrogels can be regenerated upon neutralizing with 10 N NaOH. Addition of concentrated NaOH (10 N) solution to the native hydrogel not only collapses the gel matrix but also disassemble the CT complex into a black colour solution. The stability of the hydrogel was also examined in the presence of metal ions. The addition of CaCl_2_ (1 equiv.) to the hydrogel resulted in dark brown precipitate of metal–ligand complex. The hydrogel can be reverted back from its solid state upon addition of EDTA (2 equiv.) followed by acidification to pH 6.5. Similarly, the stable CT-hydrogel transforms into viscous solution upon addition of AgNO_3_ (1 equiv.) (Fig. 5).^25^ This novel responsive NDI–Py hydrogel may act as a valuable candidate for various biomedical applications.

### 3.4 MTT assay

**Fig. 6:**
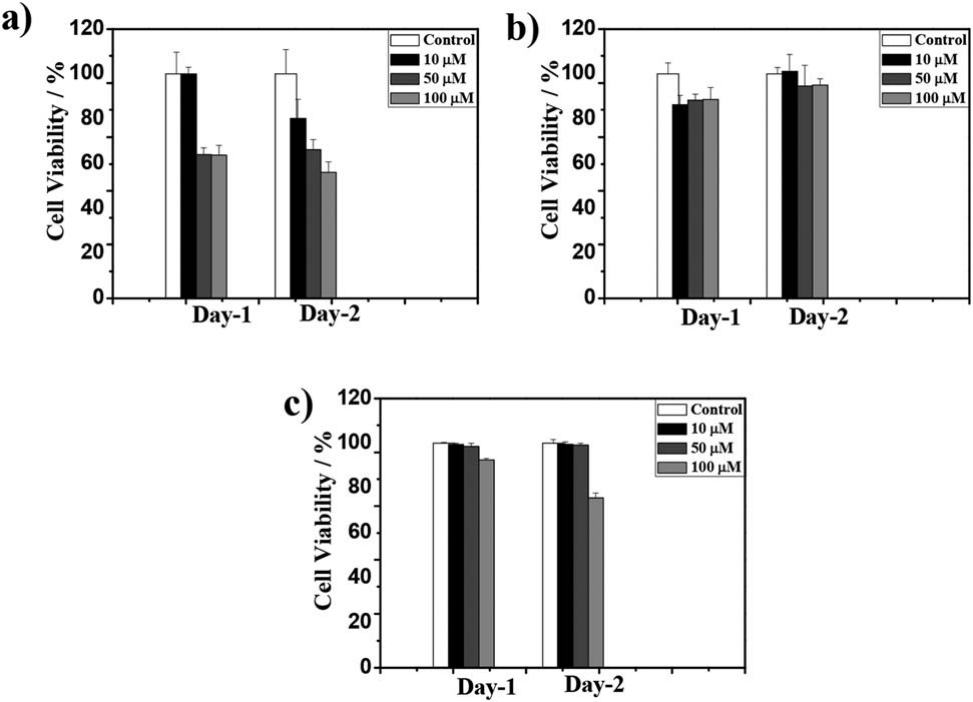
PC3 cell viability in the presence of 1 : 1 blend of (a) 1a + 2a, (b) 1d + 2d and (c), 1a + 2d at 10, 50 and 100 μM respectively.

The biocompatibility of the CT-based hydrogel has been studied with PC3 cells using a colorimetric assay with 3-(4,5-dimethylthiazole-2-yl),-2,5diphenyl tetrazolium bromide (MTT). Samples prepared at different concentrations (10, 50 and 100 μM) were tested for their proliferation capacities and the blend 1d + 2d was found to show the highest survival ratio even at 100 μM. However, the survival ratio for the 1a + 2a blend was ∼50% at 100 μM. The mismatch amino acid blend 1a + 2d also exhibited good survival ratio suitable for biological applications. All these data indicated that presence of lysine significantly reduces the cytotoxicity for PC3 cells (Fig. 6 and S10†). Furthermore, the present NDI–Py systems, particularly (1d + 2d) show low cytotoxicity and relatively good biocompatibility compare to the previously reported similar co-assembled systems.^32,45^

## 4. Conclusion

In summary, we have successfully demonstrated the self-assembly of a novel organic building blocks containing NDI and Py single amino acids. These blends undergo supramolecular co-assembly in aqueous phase *via* non-covalent interactions and exhibit deep purple colour due to the formation of characteristic CT-complex. The presence of CT interaction between donor and acceptor molecules was clearly evidenced by the various spectroscopic techniques. The NDI–Py system (1a + 2d) shows the reversible responsive behavior for various chemical stimuli. The cellular viability data show that the NDI–Py single amino acid exhibits low cytotoxicity behavior for PC3 cells. Overall, the NDI–Py mixed compounds have great potential to serve as an effective supramolecular hydrogels through the formation of CT-complex and the present system may facilitate various applications in biomedical engineering.

## Conflicts of interest

There are no conflicts to declare.

## Supplementary Material

RA-008-C8RA00929E-s001
