# Supplementary material for: The role of amino acids on supramolecular co-assembly of naphthalenediimide–pyrene based hydrogelators
Source: RSC Adv. 2018 Apr 19;8(27):14753–9. doi: 10.1039/c8ra00929e (PMC9079960; doi:10.1039/c8ra00929e)
Supplement: RA-008-C8RA00929E-s001 [file RA-008-C8RA00929E-s001.pdf]

Electronic Supplementary Material (ESI) for RSC Advances.
This journal is © The Royal Society of Chemistry 2018

The role of amino acids on supramolecular co-assembly of naphthalenediimide-pyrene based hydrogelators

Srinivasa Rao Nelli, Rajan Deepan Chakravarthy, Mohammed Mohiuddin and Hsin-Chieh Lin*

Department of Materials Science and Engineering, National Chiao Tung University, Hsinchu, 300, Taiwan, Republic of China

Supporting Information

Contents		Page Number
1.	**Synthesis of NDI and Py derivatives**	**S1-S5**
2.	**Optical images of co-assembled hydrogels**	**S6**
3.	**Hydrogelation conditions for single component system**	**S7**
4.	**Rheological data**	**S7**
5.	**Solvent dependent UV-vis absorption spectra**	**S8**
6.	**Concentration-dependent UV-vis absorption spectra**	**S8**
7.	**Temperature-dependent UV-vis absorption spectra**	**S9**
8.	**Circular Dichroism (CD) spectra**	**S10**
9.	**Solvent dependent FT-IR spectra**	**S11**
10.	**X-ray diffraction of the xerogels**	**S12**
11.	**MTT data**	**S12**
12.	**NMR spectra**	**S13**
13.	**References**	**S14**

1. Synthesis

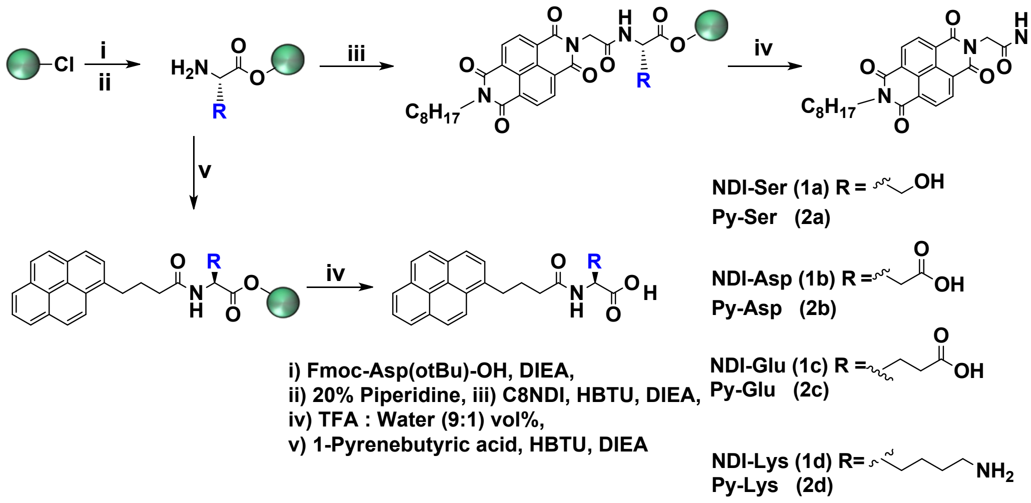

Scheme S1. Schematic representation of the synthetic route for the preparation of NDI and Py derivatives.

3.2. Preparation of NDI-derivatives (**1a-1d**):

NDI and Pyrene capped peptide derivatives ware prepared through the solid phase peptide synthesis (SPPS) using 2-chlorotrityl chloride resin and the corresponding Fmoc-amino acids.

A 1.2 g of resin was pre-swollen in anhydrous DCM for 30 min, under the N_2 conditions. Corresponding Fmoc-O-tert-Butyl-L-serine (2.000 mmol) and anhydrous N, N-Dimethlyformamide (DMF) and N, N-Diisopropylethylamine (DIEA) (5.000 mmol) were added in to the resin solution and allowed to react for 1 h. The block solution (DCM: MeOH: DIEA) was added and stirred for 30 min, followed by the addition of 20% of piperidine in DMF to de-protect Fmoc group for 30 min and repeated twice for 2 min. A 2.000 mmol of 7-octyl naphthalene diimide (C8NDI) was added to the free amino group by using O-(benzotriazol-1-yl)-N, N, N', N',-tetramethyluroniumhexafluorophospate (HBTU) 2 mmol) and N, N-Diisopropylethylamine (DIEA) (5 mmol) as the coupling reagent. Finally the reaction mixture was stirred for overnight, excessive reagents were removed by subsequently washing with

solvents. The desired compounds was achieved by treating the resine solution with 90 % trifluoroaceticacid (TFA) in DI water for 3 h. The obtained solution was collected and added diethyl for 24h, then cold diethyl ether was added to form solid. The solid was filtered and dried under the vacuum to remove the residual solvents.

3.3. Synthesis of Pyrene derivatives (**2a-2d**):

A 1.2 g of resin was pre-swollen in anhydrous DCM for 30 min, under the N_2 conditions. Corresponding Fmoc-O-tert-Butyl-L-serine (2.000 mmol) and anhydrous N, N-Dimethlyformamide (DMF) and N, N-Diisopropylethylamine (DIEA) (5.000 mmol) were added in to the resin solution and allowed to react for 1 h. The block solution (DCM: MeOH: DIEA) was added and stirred for 30 min, followed by the addition of 20% of piperidine in DMF to de-protect Fmoc group for 30 min and repeated twice for 2 min. A 2.000 mmol of 1-Pyrenebutyric acid (Py) was added to the free amino group by using O-(benzotriazol-1-yl)-N, N, N', N',-tetramethyluroniumhexafluorophospate (HBTU) 2 mmol) and N, N-Diisopropylethylamine (DIEA) (5 mmol) as the coupling reagent. Finally the reaction mixture was stirred for overnight, excessive reagents were removed by subsequently washing with solvents. The desired compounds was achieved by treating the resine solution with 90 % trifluoroaceticacid (TFA) in DI water for 3 h. The obtained solution was collected and added diethyl for 24h, then cold diethyl ether was added to form solid. The solid was filtered and dried under the vacuum to remove the residual solvents.

3.4. Synthesis of NDI-Serine (**1a**):

Dark red solid: 0.281 g; ^{1}H-NMR (300 MHz, d_6-DMSO, 25 °C): δ = 0.85-0.95 (t, *J*=7.5 Hz 3H; CH3), 1.20-1.45 (m, 10H; CH2), 1.60-1.80 (m, 2H; CH2), 3.60-3.70 (m, 1H; CH2), 3.70-3.80 (m, 1H; CH2), 4.05-4.15 (t, *J*=9.6 Hz, 2H; CH2), 4.30-4.40 (m, 1H; CH), 4.75-4.90 (s, 2H; CH2),

8.55-8.65 (d, *J*=10.4 Hz, 1H; NH), 8.65-8.75 (m, 3H; CH), 8.75-8.80 (m, 1H; CH). ^{13}C-NMR (75 MHz, d_6-DMSO, 25 °C) : δ = 14.0, 22.1, 26.5, 27.3, 28.6, 28.7, 31.3, 42.4, 54.8, 61.5, 125.7, 126.0, 126.1, 126.2, 126.5, 130.4, 130.7, 130.8, 162.3, 162.4, 166.3, 171.8. MS [ESI$^-$]: m/z (%): Calculated: 523.20, observed: 522.4 [M-H]$^-$.

3.5. Synthesis of NDI-Aspartic acid (**1b**):

NDI derivative (**1b**) was prepared in a similar manner of **1a** by the mixture of Fmoc-L-aspartic acid 4-tert-butyl ester (0.823 g, 2.000 mmol) and anhydrous N, N-Dimethlyformamide (DMF) and N, N-Diisopropylethylamine (DIEA) (0.830 mL, 5.000 mmol) were added in to the resin solution and stirred for 1 h. Light brown solid: 0.290 g; ^{1}H- NMR (300 MHz, d_6-DMSO, 25 °C): δ = 0.85-0.95 (m, 3H; CH3), 1.20-1.45 (m, 10H; CH2), 1.60-1.80 (m, 2H; CH2),1.75-2.05 (m, 2H; CH2), 2.30-2.40 (t, *J*=7.35 Hz 2H; CH2), 4.05-4.15 (t, *J*=7.2 Hz 2H; CH2), 4.25-4.35 (m, 1H; CH), 4.75-4.85 (s, 2H; CH2), 8.60-8.80 (m, 4H; CH). ^{13}C-NMR (75 MHz, d_6-DMSO, 25 °C): δ = 14.9, 23.1, 27.5, 27.6, 28.3, 29.5, 29.7, 30.9, 32.2, 43.4, 52.2, 126.7, 127.0, 127.1, 127.4, 131.4, 131.6, 163.3, 163.4, 167.4, 174.0, 174.7. MS [ESI$^-$]: m/z (%): calculated: 565.21, observed: 564.5 [M-H]$^-$.

3.6. Synthesis of NDI-Glutamic acid (**1c**):

NDI derivative (**1c**) was prepared in a similar manner of 1a by the mixture of Fmoc-L-Glutamic acid 5-tert-butyl ester (0.950 g, 2.000 mmol) and anhydrous N, N-Dimethlyformamide (DMF) and N, N-Diisopropylethylamine (DIEA) (0.830 mL, 5.000 mmol) were added in to the resin solution and stirred for 1 h. Light brown solid: 0.328 g; ^{1}H- NMR (300 MHz, d_6-DMSO, 25 °C): δ = 0.85-0.95 (m, 3H; CH3), 1.20-1.45 (m, 10H; CH2), 1.60-1.80 (m, 2H; CH2),1.75-2.05 (m, 2H; CH2), 2.30-2.40 (t, *J*=7.35 Hz 2H; CH2), 4.05-4.15 (t, *J*=7.2 Hz 2H; CH2), 4.25-4.35 (m, 1H; CH), 4.75-4.85 (s, 2H; CH2), 8.60-8.80 (m, 4H; CH). ^{13}C-NMR (75 MHz, d_6-DMSO, 25

°C): δ = 14.9, 23.1, 27.5, 27.6, 28.3, 29.5, 29.7, 30.9, 32.2, 43.4, 52.2, 126.7, 127.0, 127.1, 127.4, 131.4, 131.6, 163.3, 163.4, 167.4, 174.0, 174.7. MS [ESI^-]: m/z (%): calculated: 565.21, observed: 564.5 $[M-H]^-$.

3.7. Synthesis of NDI- Lysine (**1d**):

NDI derivative (**1d**) was prepared in a similar manner of 1a by the mixture of Nα-Fmoc-Nε-Boc-L-lysine (0.936 g, 2.000 mmol) and anhydrous N, N-Dimethlyformamide (DMF) and N, N-Diisopropylethylamine (DIEA) (0.830 mL, 5.000 mmol) were added in to the resin solution and stirred for 1 h. Light brown solid: 0.422 g; ^{1}H- NMR (300 MHz, d_6-DMSO, 25 °C): δ = 0.85-0.95 (m, 3H; CH3), 1.25-1.45 (m, 12H; CH2), 1.55-1.90 (m, 6H; CH2), 2.80-2.90 (t, *J*=10.0 Hz, 2H; CH2), 4.05-4.15 (t, J=9.6 Hz, 1H; CH2), 4.25-4.35 (m, 1H; CH) 4.75-4.85 (s, 2H; CH2), 7.70-7.80 (d, *J*=7.6 Hz, 1H; NH), 8.60-8.70 (d, *J*=10.8 Hz, 1H; CH), 8.70-8.75 (m, 2H; CH), 8.75-8.80 (m, 1H; CH). ^{13}C-NMR (75 MHz, d_6-DMSO, 25 °C): δ=14.0, 22.1, 22.4, 26.5, 26.6, 27.4, 28.6, 28.7, 30.6, 31.3, 35.9, 42.5, 51.9, 125.6, 125.87, 125.90, 126.1, 126.3, 130.4, 130.7, 162.2, 162.3, 166.4, 173.3. MS [ESI^+]: m/z (%): calculated: 564.26, observed: 565.2 $[M+H]^-$.

3.8. Synthesis of Py-Serine (**2a**):

0.162 g .^{1}H- NMR (300 MHz, d_6-DMSO, 25 °C): δ = 2.00-2.10 (m, 2H; CH2), 2.38 (t, *J*=6.4 Hz, 2H; CH2), 3.65-3.77 (m, 2H; CH2), 4.36-4.55 (m, 1H; CH), 7.95 (d, *J*=7.8 Hz, 1H; CH), 8.04-8.14 (m, 2H; CH), 8.20-8.29 (m, 6H; CH), 8.44 (d, *J*=9.3 Hz, 1H; NH). MS [ESI^-]: m/z (%): Calculated: 375.42, observed: 374.41 $[M-H]^-$.

3.9. Synthesis of Py- Aspartic acid (**2b**):

Pyrene derivative (2b) was prepared in a similar manner of 2a by the mixture of Fmoc-L-aspartic acid 4-tert-butyl ester (0.823 g, 2.000 mmol) and anhydrous N, N-Dimethlyformamide (DMF)

and N, N-Diisopropylethylamine (DIEA) (0.830 mL, 5.000 mmol) were added in to the resin solution and stirred for 1 h. 0.192 g; ^{1}H- NMR (300 MHz, d_6-DMSO, 25 °C): δ = 2.00-2.10 (m, 2H; CH2), 2.29 (t, *J*=7.2 Hz, 2H; CH2), 2.55-2.75 (m, 2H; CH2), 4.56-4.63 (m, 1H; CH), 8.00 (d, *J*=7.8 Hz, 1H; CH), 8.03-8.14 (m, 2H; CH), 8.20-8.29 (m, 6H; CH), 8.39 (d, *J*=9.9 Hz, 1H; NH). MS [ESI^-]: m/z (%): Calculated: 403.43, observed: 402.14 $[M-H]^-$.

3.10. Synthesis of Py-Glutamic acid (**2c**):

Pyrene derivative (2c) was prepared in a similar manner of 2a by the mixture of Fmoc-L-Glutamic acid 5-tert-butyl ester (0.950 g, 2.000 mmol) and anhydrous N, N-Dimethlyformamide (DMF) and N, N-Diisopropylethylamine (DIEA) (0.830 mL, 5.000 mmol) were added in to the resin solution and stirred for 1 h. 0.165 g; ^{1}H- NMR (300 MHz, d_6-DMSO, 25 °C): δ = 1.78-1.88 (m, 1H; CH2), 1.95-2.07 (m, 3H; CH2), 2.33 (m, J=7.2 Hz, 4H; CH2), 4.29 (m, 1H; CH), 7.99 (d, *J*=8.1 Hz, 1H; CH), 8.06-8.19 (m, 4H; CH), 8.23-8.32 (m, 4H; CH), 8.43 (d, *J*=9.0 Hz, 1H; NH). MS [ESI^-]: m/z (%): Calculated: 417.45, observed: 416.23 $[M-H]^-$.

3.11. Synthesis of Py- Lysine (**2d**):

Pyrene derivative (2d) was prepared in a similar manner of 2a by the mixture of Nα-Fmoc-Nε-Boc-L-lysine (0.936 g, 2.000 mmol) and anhydrous N, N-Dimethlyformamide (DMF) and N, N-Diisopropylethylamine (DIEA) (0.830 mL, 5.000 mmol) were added in to the resin solution and stirred for 1 h. 0.180 g ; ^{1}H- NMR (300 MHz, d_6-DMSO, 25 °C): δ = 1.45 (t, *J*=3.5 Hz, 2H; CH2), 1.53-1.64 (m, 3H; CH2), 1.74 (t, *J*=6.6 Hz, 1H; CH2), 2.04 (t, *J*=7.3 Hz, 2H; CH2), 2.32 (t, *J*=6.9 Hz, 2H; CH2), 2.74-2.80 (m, 2H; CH2),4.19-4.27 (m, 1H; CH), 7.68 (s, 2H; NH), 7.97 (d, *J*=7.8 Hz, 1H; CH), 8.05-8.17 (m, 4H; CH), 8.22-8.31 (m, 4H; CH), 8.41 (d, *J*=9.3 Hz, 1H; NH). MS [ESI^-]: m/z (%): Calculated: 416.51, observed: 415.24 $[M-H]^-$.

2. Optical images of a) **1a**+**2a**, b) **1b**+**2b**, c) **1c**+**2c**, d) **1d**+**2d**, e) **1a+2d** and f) **1d+2a**

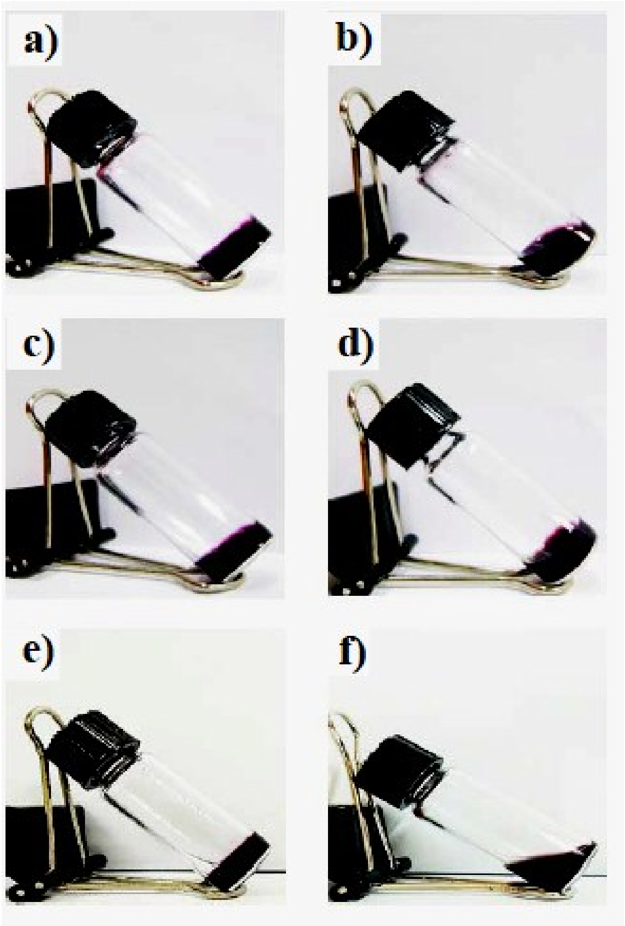

Fig. S1 Optical images of 1:1 blend of NDI-Py components a) **1a**+**2a**, b) **1b**+**2b**, c) **1c**+**2c**, d) **1d**+**2d**, e) **1a+2d** and f) **1d+2a** at 3 wt% in aqueous media.

3. Hydrogelation conditions for single components

Table S1 Correlation table for NDI and Py amino acid systems reported in the literature.

Amino acid conjugates[a]	S	D	E	K	Reference
**NDI**	**OG (pH=4.5)**	OG (pH=4.5)[b]	SG (pH=4.4)	SG (pH=10.0)	**S1**
**Py**	OS (pH=2.0-12.0)	OS (pH=2.0-12.0)	TG (pH=4.0-10.0)	**OG (pH=9.0)**	**S2**

[a]OG: opaque gel, OS: opaque solution, SG: semi-translucent gel, TG: transparent gel, [b]Data measured more recently for comparison..

4. Rheological data

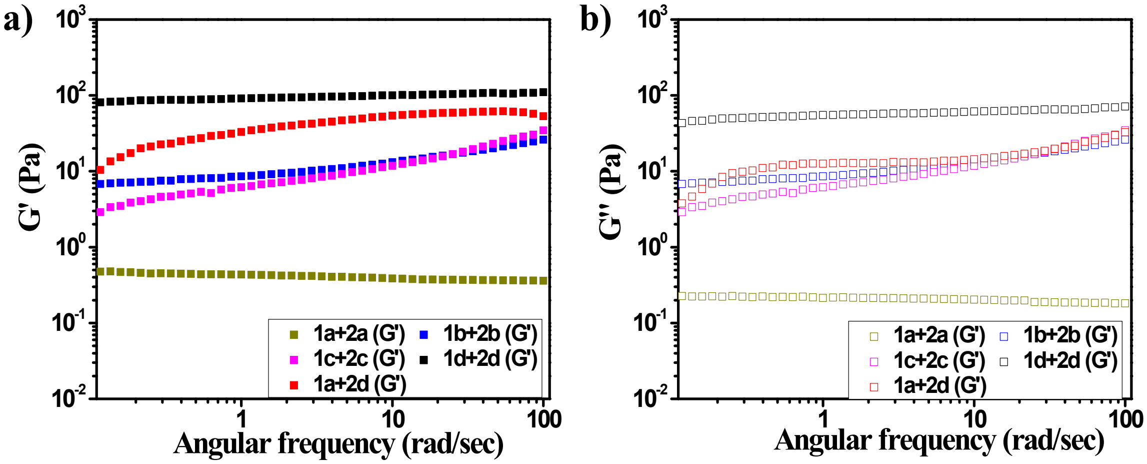

Fig. S2 The frequency sweep rheological data of 1:1 blend at 3 wt%.

5. Solvent dependent UV-vis absorption spectra

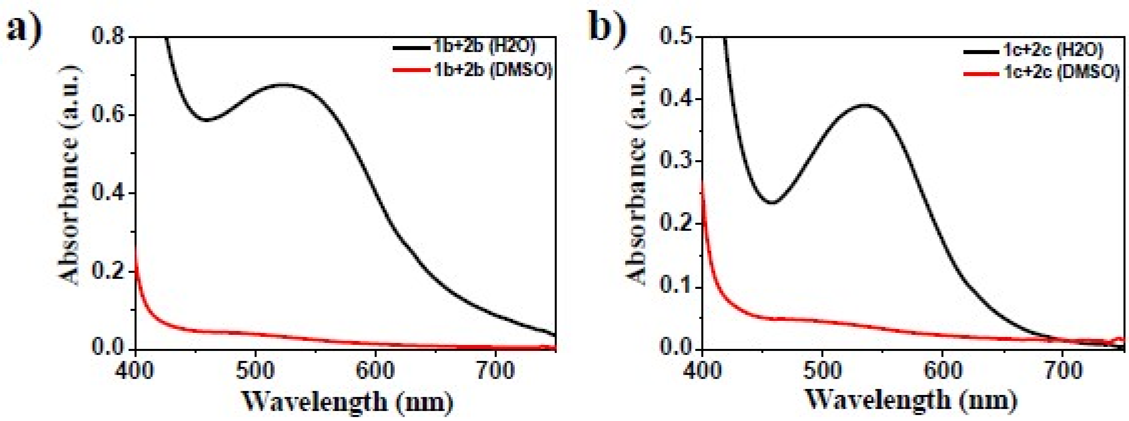

Fig. S3 Solvent dependent UV-Vis absorbtion spectra of the 1:1 blend of (a) **1b**+**2b** and (b) **1c**+**2c** (black in H_2O and red in DMSO) at 20000 µM.

6. Concentration dependent UV-vis absorption spectra

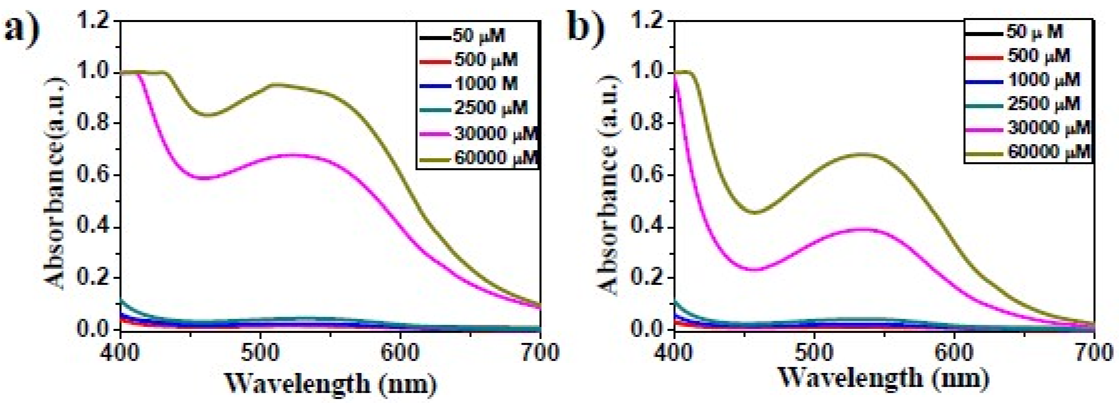

Fig. S4 Concentration dependent UV-Vis absorbtion spectra of the 1:1 blend of (a) **1b**+**2b** and (b) **1c**+**2c** at 50-60000 µM in aqueous media.

7. Temperature dependent UV-vis absorption spectra

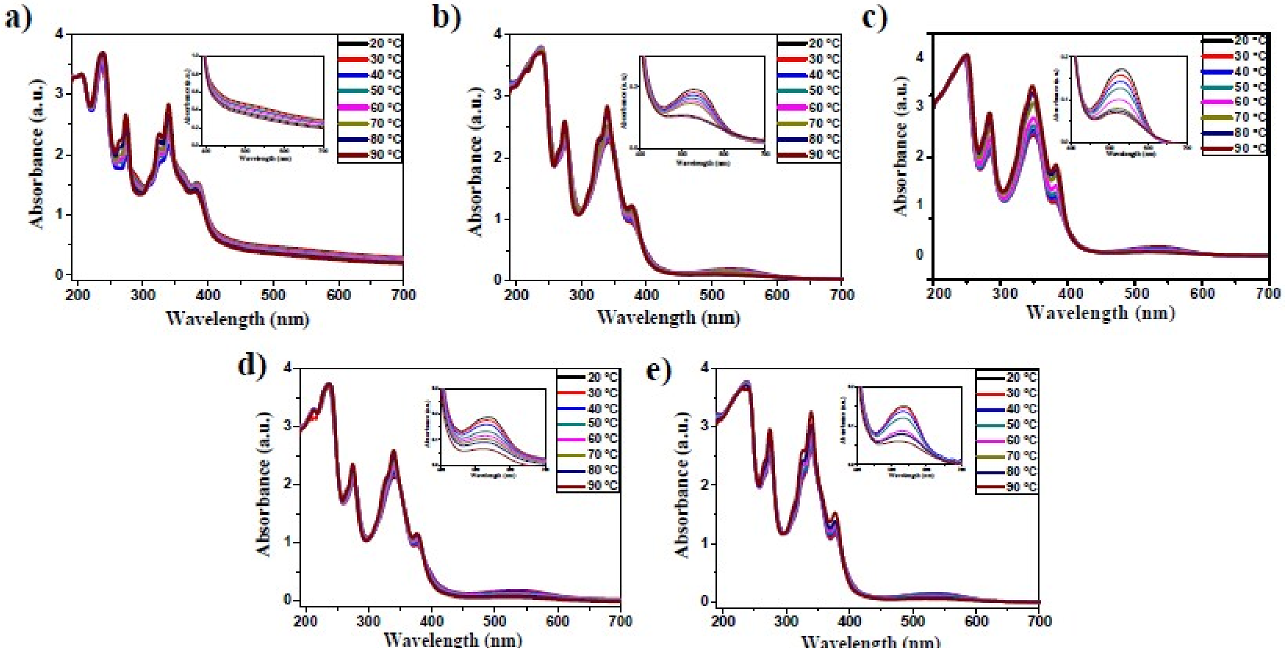

Fig. S5 Variable temperature dependent UV-vis absorption spectra of the 1:1 blend of (a) **1a**+**2a**, (b) **1d**+**2d**, (c) **1a**+**2d,** (d) **1b**+**2b** and (e) **1c**+**2c** at 20000 μM.

8. Circular Dichroism (CD) spectra

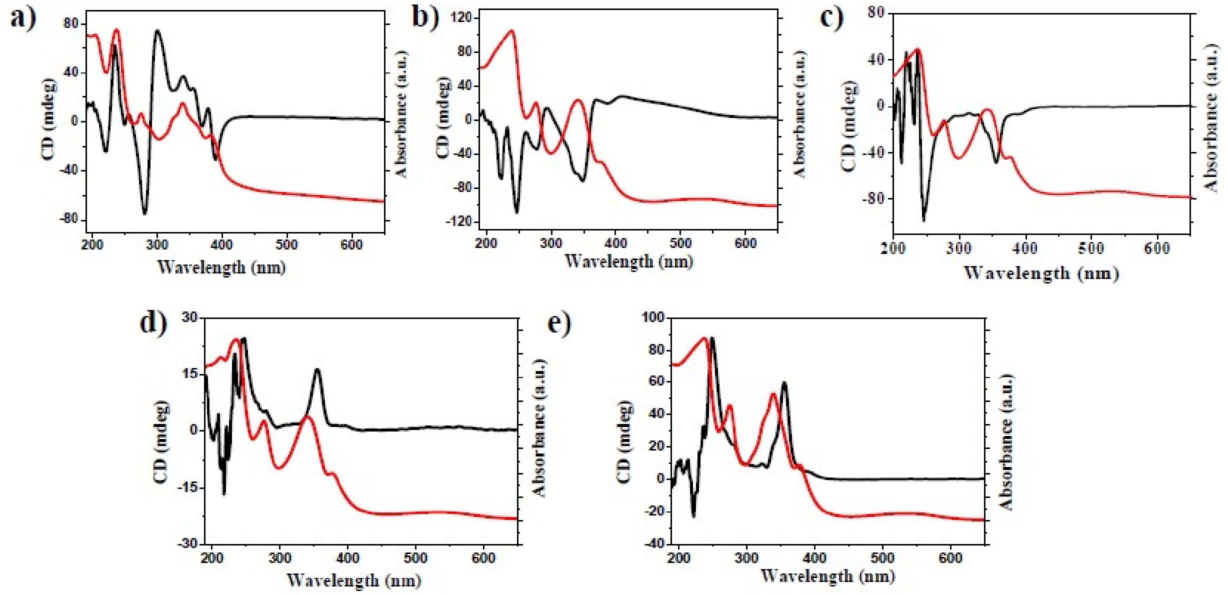

Fig. S6 UV-vis absorption spectra (red) and CD (black) of 1:1 blend of mixed components (a) **1a**+**2a**, (b) **1d**+**2d**, (c) **1a**+**2d,** (d) **1b**+**2b** and (e) **1c**+**2c** at 2500 µM in aqueous media.

9. FT-IR data

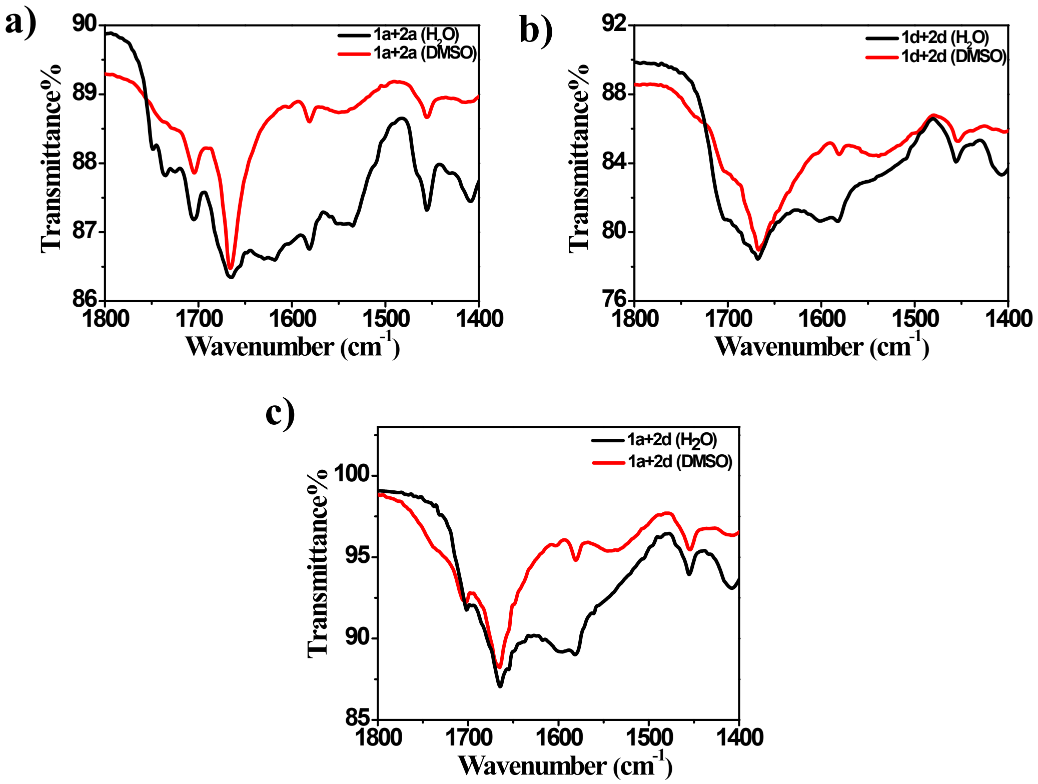

Fig. S7 FTIR spectra of (a) **1a**+**2a**, (b) **1d**+**2d** and c) **1a**+**2d** (black in H2O and red in DMSO) at 20000 μM.

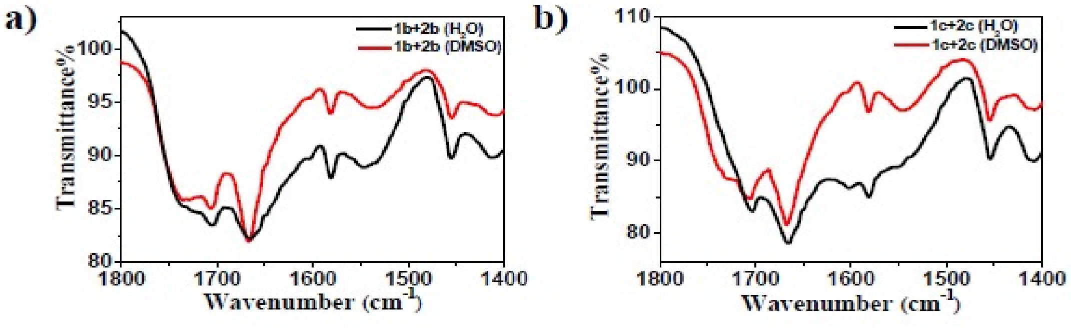

Fig. S8 FT-IR spectra of (a) **1b**+**2b** and (b) **1c**+**2c** (black in H_2O and red in DMSO) at 20000 μM.

10. X-ray diffraction of the xerogels

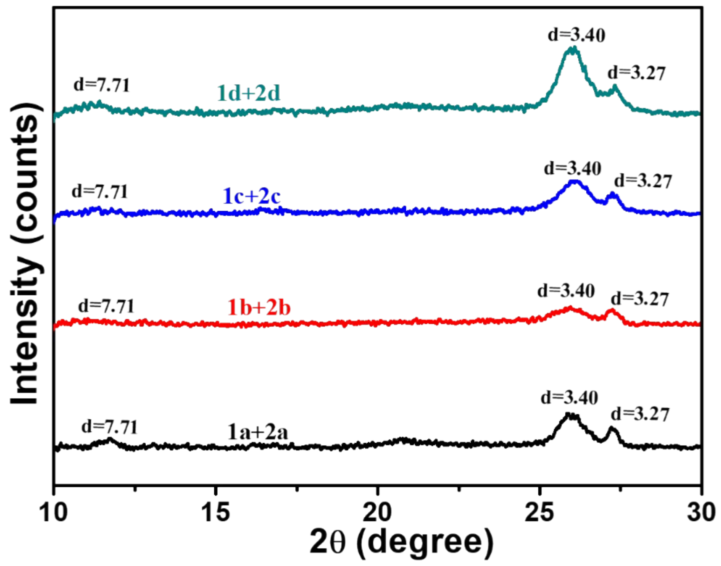

Fig. S9 Powder X-ray diffraction of the xerogels of NDI-Py gelators.

11. MTT data

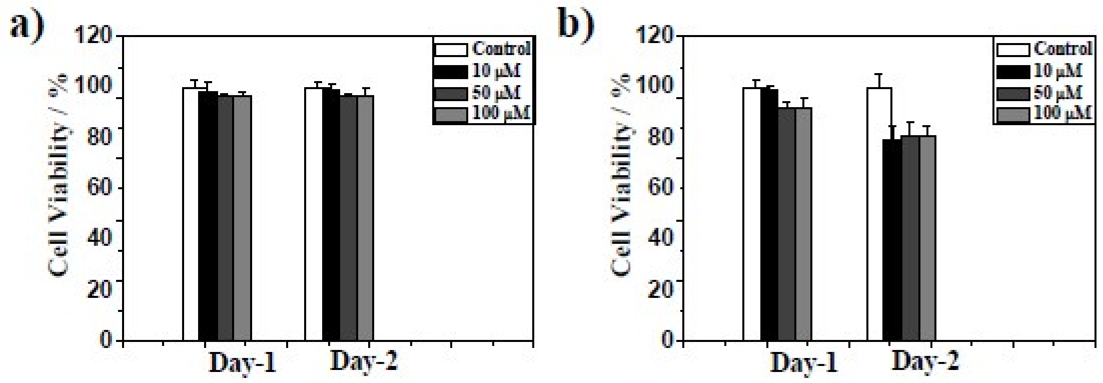

Fig. S10 PC3 cell viability in the presene of 1:1 blend of (a) **1b**+**2b** and (b) **1c**+**2c** at 10, 50 and 100 μM.

12. NMR Spectra

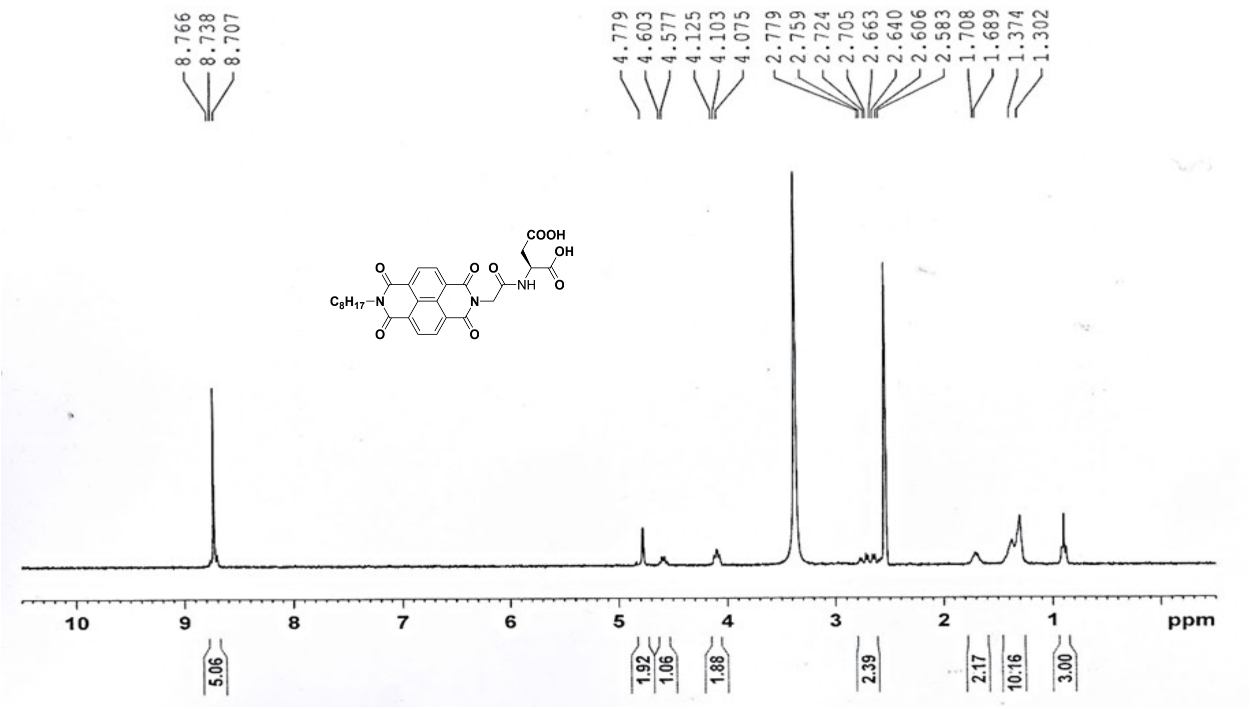

Fig. S11 ^{1}H NMR spectrum of **1b** in d_6-DMSO.

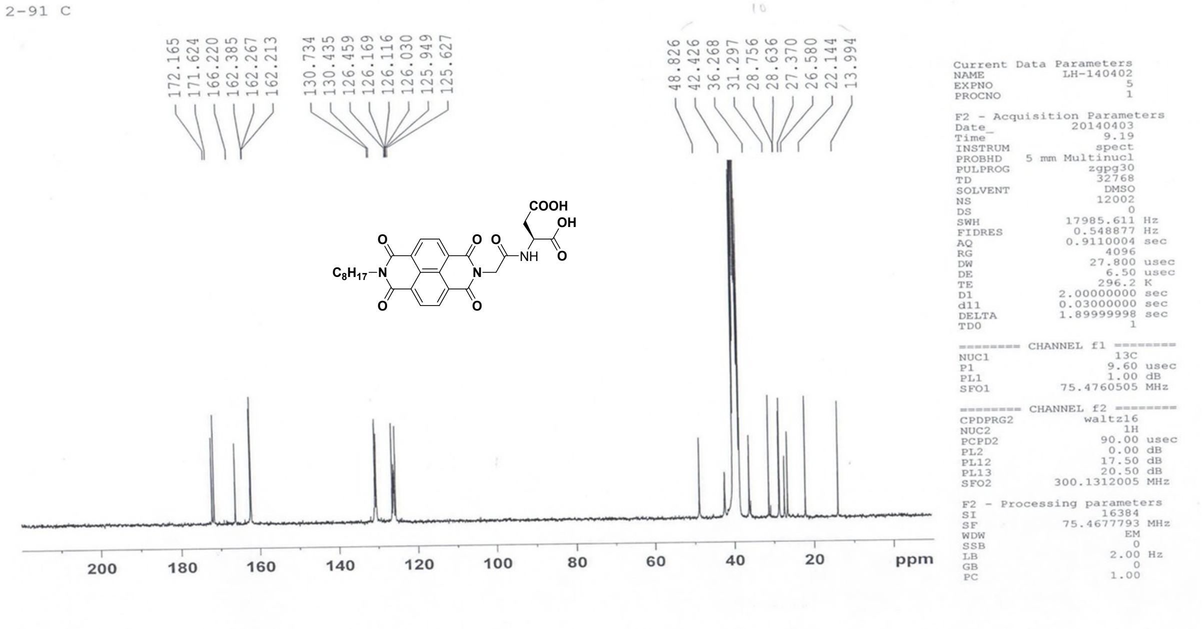

Fig. S12 ^{13}C NMR spectrum of **1b** in d_6-DMSO.

13. References

S1. S. R. Nelli, J-H. Lin, T. N. A. Nguyen, D. T-H. Tseng, S. K. Talloj and H.-C. Lin, New J. Chem. 2017, **41**, 1229.

S2. S. R. Nelli, R. D. Chakravarthy, Y.-M. Xing, J.-P. Weng and H.-C. Lin, Soft Matter, 2017, **13**, 8402.
